# Why physicians and nurses ask (or don’t) about partner violence: a qualitative analysis

**DOI:** 10.1186/1471-2458-12-473

**Published:** 2012-06-21

**Authors:** Charlene E Beynon, Iris A Gutmanis, Leslie M Tutty, C Nadine Wathen, Harriet L MacMillan

**Affiliations:** 1 Arthur Labatt Family School of Nursing, Faculty of Health Sciences, Western University, London, Canada; 2 Public Health Research, Education & Development Program, Middlesex-London Health Unit, London, Canada; 3 Department of Epidemiology and Biostatistics, Schulich School of Medicine & Dentistry, Western University, London, Canada; 4 St. Joseph’s Health Care, London, Canada; 5 Faculty of Social Work, University of Calgary, Calgary, Canada; 6 Faculty of Information & Media Studies, Western University, London, Canada; 7 Department of Psychiatry and Behavioural Neurosciences, and of Pediatrics, McMaster University, Hamilton, Canada

**Keywords:** Intimate partner violence inquiry, Barriers and facilitators

## Abstract

**Background:**

Intimate partner violence (IPV) against women is a serious public health issue and is associated with significant adverse health outcomes. The current study was undertaken to: 1) explore physicians’ and nurses’ experiences, both professional and personal, when asking about IPV; 2) determine the variations by discipline; and 3) identify implications for practice, workplace policy and curriculum development.

**Methods:**

Physicians and nurses working in Ontario, Canada were randomly selected from recognized discipline-specific professional directories to complete a 43-item mailed survey about IPV, which included two open-ended questions about barriers and facilitators to asking about IPV. Text from the open-ended questions was transcribed and analyzed using inductive content analysis. In addition, frequencies were calculated for commonly described categories and the Fisher’s Exact Test was performed to determine statistical significance when examining nurse/physician differences.

**Results:**

Of the 931 respondents who completed the survey, 769 (527 nurses, 238 physicians, four whose discipline was not stated) provided written responses to the open-ended questions. Overall, the top barriers to asking about IPV were lack of time, behaviours attributed to women living with abuse, lack of training, language/cultural practices and partner presence. The most frequently reported facilitators were training, community resources and professional tools/protocols/policies. The need for additional training was a concern described by both groups, yet more so by nurses. There were statistically significant differences between nurses and physicians regarding both barriers and facilitators, most likely related to differences in role expectations and work environments.

**Conclusions:**

This research provides new insights into the complexities of IPV inquiry and the inter-relationships among barriers and facilitators faced by physicians and nurses. The experiences of these nurses and physicians suggest that more supports (e.g., supportive work environments, training, mentors, consultations, community resources, etc.) are needed by practitioners. These findings reflect the results of previous research yet offer perspectives on why barriers persist. Multifaceted and intersectoral approaches that address individual, interpersonal, workplace and systemic issues faced by nurses and physicians when inquiring about IPV are required. Comprehensive frameworks are needed to further explore the many issues associated with IPV inquiry and the interplay across these issues.

## Background

Intimate partner violence (IPV) against women is a serious public health issue. Estimates of rates of IPV vary in the literature, depending on the definition used, data collection procedures and sampling strategies. Annual Canadian population estimates have varied over time from 10% in 1993 [1] to 1.9% in 2009 [2]. However, other surveys have found higher rates of abuse experienced by women, depending in part on the sample population. For example, in 2002–2003, Thurston and colleagues found a disclosure rate of 19.0% when women in an urgent care clinic were directly asked by nurses during the first year of implementation of a universal domestic violence screening protocol [3]. Depending on the tool used, recent Ontario, Canada trials have identified between 4.1–22% of adult women presenting to emergency departments (EDs), family medicine practices and women’s health clinics reporting IPV over the past year [4-6].

Women are not likely to disclose abuse unless directly asked [7]. Using focus groups with women who had experienced IPV and who were currently using support programs, Chang and colleagues noted that these women offered specific advice to health care providers when asking about IPV (i.e., provide a rationale for the inquiry to lessen feelings of shame and apprehension; ask when alone in a safe and supportive environment; and offer information, support and access to resources even if women do not disclose [8]). Yet, few women are asked despite presenting with signs and symptoms suggestive of exposure to IPV. A study by Glass and colleagues demonstrated that less than 25% of women presenting for any reason were asked about physical, sexual and emotional IPV by ED staff, including only 39% of women presenting with acute trauma, and 13% with past-year IPV [9]. The issue regarding whether to ask by routine universal screening, versus clinically-indicated case-finding, has been addressed elsewhere [6,10]. Recent randomized trial level evidence does not support universal screening [4,11].

Barriers and facilitators to asking about IPV vary according to a number of factors. For example, Rodriguez and colleagues surveyed 375 culturally diverse women who attended public clinics to examine factors associated with abuse disclosure to physicians [12]. Forty-two percent of these women stated they had talked to a physician about their abuse. These respondents perceived that physicians did not ask directly about abuse and that they had insufficient time and interest in discussing abuse. Furthermore, respondents described fear of legal involvement and concerns about confidentiality as barriers to disclosing abuse. However, of all the factors measured in this study, clinician inquiry was the strongest determinant of abuse disclosure.

Other studies have focused on the experiences of health care professionals. In an ethnographic study of 38 physicians who were primarily family practitioners working in an urban health maintenance organization, barriers to addressing IPV included lack of comfort, fear of offending, powerlessness, frustration, loss of control and time constraints [13]. Similarly, a review article indicated that lack of provider education regarding woman abuse, fear of offending women, lack of time, clients not disclosing and lack of effective interventions were important barriers to asking about IPV [14]. Rodriguez et al. also noted that physicians were more likely to identify patient-related barriers to identification and intervention than physician-related barriers [15].

Similar issues were identified by nurses, including lack of time, lack of training in both assessment and how to respond as well as unique challenges related to their role including pressure from physicians to see patients quickly, presence of family members, language and cultural differences and challenges of screening older clients and clients with mental health issues [16-18]. Other studies of health care practitioners have found that older, more experienced clinicians, and those with histories of exposure to abuse, were more likely to ask about IPV [19].

### Study objective

The overall objective was to identify barriers and facilitators to asking about IPV among a large, randomly selected sample of nurses and physicians in specified areas of practice where abused women are likely to present. Specific goals were to: 1) explore physician’s and nurses’ experiences, both professional and personal, when asking about IPV; 2) determine variation by discipline i.e., nurse or physician; and 3) identify implications for practice, workplace policy and curriculum development. This study was part of the McMaster Violence Against Women Research Program, a multi-study program of research developed to investigate the health care response to IPV experienced by women, including how best to identify women presenting to health care settings.

## Methods

### Sample and data collection

This paper summarizes responses to two open-ended questions about barriers and facilitators to asking about IPV: 1) What do you experience as barriers to screening for woman abuse?; and 2) What has helped or would help make screening for woman abuse easier for you? These were the final questions on a mailed self-administered 43-item survey that addressed barriers and facilitators to asking about IPV and also included questions related to respondent demographics, education, training, and professional and personal experience with IPV. The survey was mailed to a random sample of 1,000 physicians, weighted by specialty (family practice, emergency medicine, public health, obstetrics and gynaecology) and an unweighted random sample of 1,000 nurses working in family physician offices, emergency care, maternal/newborn and public health. The data were collected from March to June 2004 and analysis of the qualitative data occurred from 2004 until 2009.

A modified Tailored Design Method was used to enhance the response rate [20]. Potential respondents received an advance notice advising them of the survey; one week later, the 1,000 nurses and 1,000 physicians were sent a personalized letter of information, the survey and a token gift certificate for an Ontario-wide coffee shop. A reminder letter and a replacement survey were mailed to all potential participants three weeks later. A complete description of the survey development, sampling and methods is available in our previous publication of the quantitative results [21].

### Data management and analysis

Responses to the two open-ended questions were entered into a word processing document. To ensure data quality, one of the study investigators reviewed all transcribed responses; any questionable responses were identified and another investigator checked these responses with the original submission. Inductive content analysis was used as the method of analysis; that is patterns and categories emerged from the data and were not decided upon before the analysis [22]. The investigators independently examined the transcribed data for common categories regarding barriers and facilitators by identifying key words, phrases or concepts used by respondents. The project team vetted the resulting categories. The first author then coded the written responses by discipline i.e., nurse or physician. As each category was analyzed, attention was given to similar and contrasting perspectives, as well as unique viewpoints. Random checks were performed by the second author to ensure that the coding was consistent and that the frequencies identified by categories accurately reflected the responses. Those few coding discrepancies that were identified were resolved through dialogue.

Frequencies were calculated for the commonly identified categories and because of the relatively low number of observations in some of the two-by-two tables, the Fisher’s Exact Test was performed to determine statistical significance when examining differences between nurses and physicians [23]. Although such an approach to data analysis is infrequent when conducting qualitative analyses, it does reflect the positivist epistemology that underpinned development of the self-administered survey tool [24,25].

The intent of the analysis for this paper was to focus on differences between nurses and physicians and did not include an analysis by practice setting. Some exceptions where practice settings are identified are noted. Ethics approval was obtained from the Research Ethics Board of Western University, London, Canada.

## Results

In total, 931 individuals returned completed questionnaires; 597 identified themselves as nurses (59.7% response rate) and 328 indicated that they were physicians (32.8% response rate). Six respondents did not specify their discipline. Of the total 931 responses, 769 (82.6%) provided written comments to the two open-ended questions, i.e., 527 nurses (88.3% of nurse respondents), 238 physicians (72.6% of physician respondents) and four who did not identify their discipline. Table 1 shows the demographic characteristics, training and professional/personal experience of those who provided written comments. This group is very similar to the larger sample of 931 in terms of gender, age, area of practice, formal training and professional and personal experience with woman abuse. Consistent with the full study sample, the majority of those providing comments were female (81.1%, n = 624), which was primarily driven by the nursing data. Of those physicians who provided comments, the data were fairly evenly split by gender, with slightly more male (53.4%, n = 127) than female (46.6%, n = 111) physicians offering written comments. Furthermore, of those who provided written comments, the majority of nurses (61.5%, n =324) and physicians (58.0%, n = 138) had not received formal IPV training and the majority of both nurses and physicians experienced less than 20 disclosures in the past year.

**Table 1:** Demographic characteristics of study sample

	**Full study sample (931)**	**Sample providing comments (769)**	Nurses with comments (527)*	Physicians with comments (238)*
**Sex**				
Female	77.6% (722)	81.1% (624)	97.2% (512)	46.6% (111)
Male	21.8% (203)	18.1 (139)	2.3% (12)	53.4% (127)
Missing	0.6% (6)	0.8% (6)	---	0
**Age (years)**				
20–29	6.9% (64)	7.4% (57)	9.7% (51)	2.1% (5)
30–39	24.9% (232)	25.0% (192)	24.1% (127)	27.3% (65)
40–49	33.1% (308)	32.9% (253)	32.1% (169)	35.3% (84)
50–59	28.8% (268)	27.7% (213)	27.7% (146)	28.2% (67)
60+	5.6% (52)	6.1% (47)	5.7% (30)	7.1% (17)
Missing	0.8% (7)	0.9% (7)	---	0
**Current area of practice**				
Family medicine	32.2% (300)	29.3% (225)	8.7% (46)	74.8% (178)
Emergency medicine	21.2% (197)	21.7% (167)	27.1% (143)	9.7% (23)
Public health	17.8% (166)	19.0% (146)	26.9% (142)	*---*
OB/gyn/newborn	22.6% (210)	23.8% (181)	30.7% (162)	8.8% (21)
Retired + other	4.0% (37)	4.0% (31)	5.3% (28)	---
Missing	2.3% (21)	2.2% (17)	1.1% (6)	4.6% (11)
**Any disclosure**				
Never	23.1% (215)	22.6% (174)	30.4% (160)	5.0% (12)
None this year	10.3% (96)	10.4% (80)	12.1% (64)	6.7% (16)
Less than 20 this year	63.1% (587)	63.1% (485)	54.1% (285)	83.2% (198)
20 or more this year	2.5% (23)	2.9% (22)	2.1% (11)	4.6% (11)
Missing	1.1% (10)	1.0% (8)	1.3% (7)	---
**Formal intimate partner violence (IPV) training**				
No	61.5% (573)	60.3% (464)	61.5% (324)	58.0% (138)
Yes	36.7% (342)	38.2% (294)	37.0% (195)	40.8% (97)
Missing	1.7% (16)	1.4% (11)	1.5% (8)	---
**Respondent, friend of relative experience**				
No	49.7% (463)	46.4% (367)	42.7% (225)	54.2% (129)
Yes	48.4% (451)	52.1% (401)	55.6% (293)	45.0% (107)
Missing	1.8% (17)	1.4% (11)	1.7% (9)	---

**Note**: * 4 did not indicate if they were a physician or a nurse; the number in the parenthesis is the sample size; ---: proportion suppressed, based on fewer than 5 observations; OB/gyn/newborn: Obstetrics/gynaecology/care of newborns.

While some responses were a single word or a brief phrase, other respondents provided several sentences or a paragraph. Respondents offered both professional and personal experiences, shared their opinions and asked questions. Quotations provided in the text have been purposefully selected to reflect common themes and experiences of both physicians and nurses across practice settings.

### Barriers to asking about IPV

Analysis of the data from the open-ended questions identified nine categories of barriers (Table 2). Overall, the barriers described most often by nurses and physicians were lack of time, behaviours attributed to women living with abuse and lack of training. The top five barriers described by nurses were lack of time (27.3%), lack of training (20.9%), behaviours attributed to women living with abuse (19.9%), partner presence (19.5%) and language/cultural practices (18.4%) while the top five barriers described by physicians were lack of time (46.2%), behaviours attributed to women living with abuse (25.2%), lack of resources (18.9%), language/cultural practices (8.8%) and lack of training (5.5%). Table 3 summarizes the top five barriers by discipline.

**Table 2:** Barriers to asking about IPV described by nurses and physicians (n = 769)

**Category**	**All Respondents (n = 769)**	**Nurses (n = 527)**	**Physicians (n = 238)**
1 Lack of time^a^	33.0% (254)	27.3% (144)	46.2% (110)
2 Behaviours attributed to women living with abuse	21.5% (165)	19.9% (105)	25.2% (60)
3 Lack of training^a^	16.4% (126)^b^	20.9% (110)	5.5% (13)
4 Language/cultural practices^a^	15.5% (119)^c^	18.4% (97)	8.8% (21)
5 Partner presence^a^	14.0% (108)	19.5% (103)	2.1% (5)
6 Lack of resources^a^	13.0% (100)	10.4% (55)	18.9% (45)
7 Lack of space/privacy^a^	8.2% (63)	11.2% (59)	1.7% (4)
8 Discomfort with topic	4.6% (35)	4.7% (25)	4.2% (10)
9 Lack of practitioner knowledge of resources	2.6% (20)	2.7% (14)	2.5% (6)

**Note**:

*IPV* Intimate Partner Violence; ^a^ significant differences between nurses and physicians, Fisher Exact Test, p≤ 0.005; ^b^ three respondents did not identify discipline; ^c^ one respondent did not identify discipline.

**Table 3:** Top five barriers by discipline

**Nurses (n = 527)**	**Physicians (n = 238)**
1 Lack of time (27.3%)	1 Lack of time (46.2%)
2 Lack of training (20.9%)	2 Behaviours attributed to women living with abuse (25.2%)
3 Behaviours attributed to women living with abuse (19.9%)	3 Lack of resources (18.9%)
4 Partner presence (19.5%)	4 Language/cultural practices (8.8%)
5 Language/cultural practices (18.4%)	5 Lack of training (5.5%)

Two physicians used the metaphor of *opening up a can of worms* when describing barriers. This analogy likely highlights the multi-faceted complexities faced by practitioners and the potential challenges that the respondents may have experienced when asked how much they agreed with the statements on the 43-item mailed survey regarding possible barriers to asking about IPV. Further details regarding barriers are profiled below.

#### Lack of time

As shown in Table 2, lack of time was cited as the most frequent barrier by both nurses (N) and physicians (P), yet physicians described this barrier significantly more often than nurses (physicians: 46.2%; nurses 27.3%).

One physician noted:

"*Tend to be lengthy issues…therefore very difficult to address these with the time constraints of a busy ER* [Emergency Room]*. Also I will never see the patient again so difficult to develop appropriate patient/doctor relationships with such a major issue in a brief time period.* (P)"

Often, lack of time was associated with other factors such as heavy workloads, the time required to adequately deal with the issue and the demands of a busy workplace as this nurse shared:

"*Time issues. If you are going to ask, you have to have the time to listen to the response and deal with the issue.* (N)"

Another nurse also wrote about the personal impact:

"*We have very little time for patient care. Emotional support is not always there, heavy workload and fast paced environment. I walk away from a shift feeling I really have not done the best job for some of the women.* (N)"

#### Behaviours attributed to women living with abuse

Similar proportions of physicians (25.2%) and nurses (19.9%) described the behaviours attributed to women living with abuse as a barrier. Some respondents expressed a sense of frustration if a woman had disclosed abuse and then stayed or returned to the abusive partner. This was especially challenging when the practitioner had invested effort in developing a relationship as this physician wrote:

"*I have been repeatedly frustrated by women who, after I have taken the trouble to provide alternatives for them, have ‘backed down’ and returned to their abusive partners.* (P)"

Others seemed to be more confused than frustrated by such behaviour as this nurse shared:

"*I have heard women say ‘but I love him’. I don’t understand that thinking especially when children are involved.* (N)"

However, others appeared more judgmental and critical as evident by the following responses:

"*I find they defend their partner or don’t want what I am offering.* (N)"

"*They don’t disclose and are reluctant or unable to help themselves.* (P)"

"*Certain clients don’t talk and refuse to do anything about it.* (P)"

In contrast, a physician wrote the following comment capturing the secrecy and the layers of complexity associated with IPV:

"*Some women have great ‘masks’- trying to peel off these ‘masks’ so as to see the true problem can be difficult.* (P)"

Some respondents indicated that such a lack of action by abused women could be linked to feelings of social stigma, fear, a personal sense of embarrassment or failure and a perception that the abuse is deserved. Seven nurses from different practice settings specifically commented that women might deny abuse since they were concerned that a disclosure could result in the apprehension of their children by child protective services. One nurse provided the following comment about the impact on her practice of her interpretation of the legislation regarding her duty to report situations where children are perceived to be at risk of abuse to an Ontario child protective agency (generally referred to as the Children’s Aid Society or CAS):

"*I feel****obliged****to tell women that if they****now****disclose violence****we****must call CAS. I think that this is a****huge****barrier to what we are trying to achieve- safety of the woman and in this way safety of the child. I believe many health workers have changed practice due to this legal requirement… I believe this law has set this issue back 10 years.* (N) - emphasis in original"

This nurse’s description of her experiences captures the complexities of IPV inquiry and reflects that IPV is not owned solely by the health care sector but rather requires links with multiple sectors including child welfare and law enforcement. This written response also profiles the internal conflicts encountered by practitioners when balancing professional expectations and the personal face of IPV with a desire to avoid potentially undesirable consequences and making the situation worse for the woman. There is also a sense of a “conveyor belt” syndrome that once events are set in motion there is no turning back.

#### Lack of training

A significantly lower proportion of physicians (5.5%) than nurses (20.9%) described a lack of training as a barrier. Nevertheless, as evident by the following written responses, some nurses and physicians were very open about their lack of training and knowledge often linking the need for additional training to fears of offending women, wanting to know how and when to initiate the topic and what to do following disclosure:

"*Knowing when and where to ask, without offending the woman.* (N)"

"*Not being afraid to ask. Being candid - ask questions simply and do not tread on eggshells.* (P)"

#### Partner presence

The abusive partner’s tendency to stay by the woman’s side was described as a barrier significantly more by nurses (19.5%) than by physicians (2.1%). Nurses wrote that partner presence made it especially challenging to interview the woman alone. This observation was not limited to the hospital sector and was noted for home visits, prenatal and ED visits as well as for labour and delivery.

#### Lack of space and privacy

Significantly more nurses (11.2%) than physicians (1.7%) described lack of space and privacy as barriers. This included the presence of other family members as well as other patients and work environments that were not conducive to confidential one-on-one interviews. Considering both partner presence and lack of space and privacy suggest that these are substantial barriers for nurses.

#### Language and cultural practices

A significantly greater percentage of nurses (18.4%) than physicians (8.8%) cited language and cultural practices as barriers. Frequently, the written responses from both nurses and physicians for this barrier were brief (e.g., *cultural differences/language)*. Others provided more descriptive comments as evident by the following:

"*Many women in the practice where I work would not admit to physical or mental abuse as it appears to be accepted in their culture and no amount of questioning will get them to admit it.* (N)"

"*Cultural difficulties- fear of inadequate protection from the police. Therefore many instances, women will decline anything more than just reporting to me.* (P)"

#### Lack of quality resources

A significantly greater percentage of physicians (18.9%) than nurses (10.4%) described the lack of quality resources as a barrier as evident by the following responses:

"*Ability to get help and treatment quickly and safely.* (P)"

"*No real effective support both in psychosocial and legal.* (P)"

"*Once we have identified the problem, there are serious dangers in women leaving the situation and not enough community resources to help them if family and friends cannot help them.* (P)"

"*In the North there are no resources or ways out for these women and I spent a lot of time listening to them. That was all we could provide and it was quite sad.* (N)"

Confidential and culturally sensitive interpreters and other specialized services for immigrants were described as resources that were often missing. Although lack of resources was described as a barrier, very few physicians and nurses described lack of knowledge of resources as a barrier (physicians 2.5%; nurses 2.7%).

#### Rural settings

Issues related to providing care in rural settings were described by a few respondents as a barrier to asking about IPV. One physician referred to *lack of resources in small rural setting. [sic] [s]mall town attitude*, while another physician described the small community as a facilitator: *I often know of people’s problems before they come to me.* Another respondent seemed to question the perception that urban centres have better resources.

"*I work in an urban ER and it’s great to be able to identify and approach women at risk but it seems that (sometimes) the support system is not available to help her when she needs it.* (N)"

#### Personal discomfort

Very few respondents described their own personal discomfort with IPV as a barrier (nurses 4.7%; physicians 4.2%).

#### Inadequate management support

In addition to the barriers noted in Table 2, several nurses referenced inadequate management support as a barrier.

"*Administrators who do not see the importance of this as a social broad determinant of health issue - concerned about backed up clinic schedules and the cost of time involved*. (N)"

"*I feel very comfortable screening and do screen in my scope of practice, however this is not supported by my manager in the agency I am employed by. Therefore, we do not have a consistent tool and do not receive training. (N)*"

"*Many abusers complain about the nurses and care and we have little support from management. We end up having to write up the situations. The fear of being reported as more complaints are being sent to the CNO (*College of Nurses of Ontario) *is now a factor.*(N)"

Recognizing the differences in employment models, organizational structures and reporting relationships experienced by nurses and physicians, it is not surprising that this barrier was described by nurses and not physicians. These experiences suggest that management plays a critical role in supporting or hindering IPV inquiry by nurses.

In summary, as noted in Table 2, there were statistically significant differences between nurses and physicians regarding the following barriers: lack of time; lack of training; language/cultural practices; partner presence; lack of resources; and lack of space/privacy.

### Facilitators to asking about IPV

Analysis of the data from the open-ended questions identified eight categories of facilitators, some of which differed by discipline (Table 4). The top facilitators for nurses were training (47.8%), community resources/professional supports (25.2%) and professional tools/protocols/policies (20.3%), while the most frequent facilitators reported by physicians were community resources/professional supports (21.8%), training (17.6%) and client educational materials (13.0%). Table 5 summarizes the top five facilitators by discipline. Further details regarding facilitators are outlined below.

**Table 4:** Facilitators to asking about IPV described by nurses and physicians (n = 769)

**Category**	**All respondents (n = 769)**	**Nurses (n = 527)**	**Physicians (n = 238)**
1 Training^a^	38.5% (296)^b^	47.8% (252)	17.6% (42)
2 Community resources/professional supports	24.1% (185)	25.2% (133)	21.8% (52)
3 Professional tools/protocols/policies^a^	17.3% (133)	20.3% (107)	10.9% (26)
4 Client educational materials^a^	8.6% (66)	6.6% (35)	13.0% (31)
5 Routine screening	8.6% (66)	9.5% (50)	6.7% (16)
6 Having time	6.2% (48)	6.5% (34)	5.9% (14)
7 IPV experience: professional & personal	6.1% (47)	7.2% (38)	3.8% (9)
8 Societal awareness	6.0% (46)	6.1% (32)	5.9% (14)

**Note**:

*IPV* Intimate Partner Violence; ^a^ significant differences between nurses and physicians, Fisher Exact Test, p≤ 0.005; ^b^ two respondents did not identify discipline.

**Table 5:** Top five facilitators by discipline

**Nurses (n = 527)**		**Physicians (n = 238)**	
1	Training (47.8%)	1	Community resources/professional supports (21.8%)
2	Community resources/professional supports (25.2%)	2	Training (17.6%)
3	Professional tools, protocols/policies (20.3%)	3	Client educational materials (13.0%)
4	Routine screening (9.5%)	4	Professional tools/protocols/policies (10.9%)
5	IPV experience: professional & personal (7.2%)	5	Routine screening (6.7%)

#### Training

A significantly greater percentage of nurses (47.8%) described training as a facilitator in comparison to physicians (17.6%). Overall, the respondents clearly described how they wanted to learn about IPV. The approaches described included: receiving literature and written material; developing and disseminating “best practices”; using “real-life” scenarios; role playing; talking with colleagues; having opportunities to practice newly learned skills with others who have greater comfort and experience with the topic; having discussions with women who are survivors; and touring women’s shelters. Two nurses commented on the responsibility of the employer to offer training at the worksite, while another acknowledged the challenges of shift work and being able to participate in educational sessions.

#### Community resources and professional supports

Both physicians (21.8%) and nurses (25.2%) cited the availability of community resources and professional supports as facilitators for them as practitioners. Examples described as facilitators included: inventories of local resources; access to colleagues for consultation; being part of a multidisciplinary team; and the availability of staff with specialized expertise for consultation. References to other disciplines or services focused primarily on social workers and Children’s Aid Society staff. Social workers clearly emerged as playing a critical role, one respected by both physicians and nurses. Despite the favourable comments regarding the role played by social workers, not all practitioners felt supported 24/7*.*

#### Professional tools, protocols and policies

Significantly more nurses (20.3%) described tools, protocols or policies as facilitators to support screening as a facilitator, whereas this was described by only 10.9% of physicians. Routine screening was mentioned by 9.5% of nurses and 6.7% of physicians and was believed to make asking about IPV easier. Some respondents were working in settings where this was an expectation and others recommended that routine screening be instituted. These results included 15 nurses who specifically recommended that screening be *mandatory/required/obligatory* and one physician, who advocated that screening be *recommended by law*. In contrast, one nurse recommended strongly against universal screening.

"*It is an unrealistic expectation to be screening every woman/female over the age of 12 for abuse on each encounter with health care personnel as the guidelines*[26]*have suggested. This expectation turned many emergency room nurses against screening when it was first introduced and compliance remains very low.* (N)"

#### IPV experience

Both professional and personal experiences were described by 7.2% of nurses and 3.8% of physicians as being facilitators to asking about IPV. Thirteen nurses and one physician shared that they had personally experienced abuse in their own lives and that this experience enhanced their capacity to work with abused women as evident by the following written comments:

"*My personal experience with abuse provides me with a comfort level, knowledge of the system and a desire to support and empower women.* (N)"

"*The fact that I have been a victim of domestic violence and abuse makes it easier for me to identify women who are experiencing a similar situation.* (N)"

"*My own experience has helped. Having the time in my own life to deal with my own issues has helped the most. I cannot imagine that you can teach this to someone - it is so intricate and complicated.* (P)"

One nurse who acknowledged her inexperience with abuse described how she relied on a colleague who had personally experienced abuse.

#### Educational materials

A significantly greater percentage of physicians (13.0%) described educational materials for women as a facilitator, while this was the case for only 6.6% of nurses. This difference may be accounted for by the difference in roles, where the physician was more likely to experience disclosures and depending on the practice setting assume responsibility for initiating care.

Some respondents acknowledged an increased awareness of IPV in the larger community, yet were concerned that more awareness is needed. Only 6.1% of nurses and 5.9% of physicians described societal awareness as a facilitator. As an example, one nurse commented: *It will always remain a serious problem until more information e.g. ads, commercials etc. show women it is not their fault.*

Public awareness campaigns, as well as community forums and meetings, were recognized for the key role they play in promoting awareness. Several respondents referred to the role that the media, such as television, radio and print play. The media was recognized for its critical role in promoting awareness of both IPV as well as available community services. Furthermore, the media was seen as a mechanism to normalize routine questioning by practitioners regarding abuse, so women are not surprised or offended by such questions. Mandatory education in schools was also recommended as a strategy to enhance greater societal awareness.

#### Time

Consistent with the finding that lack of time was the most frequently described barrier by both physicians (46.2%) and nurses (27.3%), only 5.9% of physicians and 6.5% of nurses cited having time as a facilitator.

In summary, Table 4 highlights several statistically significant differences between nurses and physicians regarding the following facilitators: training; professional tools/protocols/policies; and client educational materials.

## Discussion

The majority of 931 physicians and nurses completing a survey regarding their experience with IPV inquiry provided written responses to open-ended questions. These questions may have encouraged respondents to reflect and share their personal experiences. Perhaps respondents were also indicating that the short statements regarding barriers and facilitators included in the 43-item survey were too simplistic and did not fully capture the complexities of the issues they face related to IPV.

Overall, the top barriers to asking about IPV were lack of time, behaviours attributed to women living with abuse, lack of training, language/cultural practices and partner presence, while the facilitators cited most often included training, community resources/professional supports, and professional tools/protocols/policies. The statistically significant differences between nurses and physicians regarding both barriers and facilitators are most likely related to differences in roles and work environments.

After reviewing the comments, the study investigators were left with the impression that this is a very emotionally charged and complex practice issue for both nurses and physicians. Many nurses and physicians in this study continued to struggle with IPV inquiry. The sharing of personal stories – even in this self-administered, written format, was especially poignant and a reminder of additional burdens that some practitioners face related to IPV.

The two images of *peeling off a mask* and *opening a can of worms* further suggest the perceived complexities of this issue. The analogy of opening up a can of worms (or a “Pandora’s Box”), also noted by Sugg and Inui [13] and McCauley and colleagues [27] implies a sense of unpredictability and concern about having the necessary time and skills to deal with the many issues associated with abuse inquiry and disclosure. It is possible that for those who operate from a results-driven model of care, where actions are expected to solve problems, the inability to control a situation or the outcome, can be personally and professionally intimidating or frustrating. Understanding and accepting the lived experience of abused women, which may challenge practitioner logic, can be emotionally draining, while customizing care to unique circumstances and searching out resources may be time consuming and challenging. The image of peeling off a mask illustrates the challenges faced by some practitioners. It highlights the energy and time required of practitioners, the intensity of the experience for both abused women and practitioners and the secretiveness of IPV as a societal and practice issue. Such metaphors challenge us to explore approaches at multiple levels (i.e., practitioner, practice setting, workplace and community) and to ensure that practitioners have the necessary skill sets as well as on-going education and supports in their work environment.

Given that dealing with complex practice issues, is by definition, typically time consuming, the authors were not surprised that lack of time was the most frequently described barrier for nurses and physicians. Insufficient time is often cited as a barrier for a variety of practice issues. Lack of time is an important factor in health care environments, yet it can be a quick and almost automatic, impersonal response when asked to identify possible barriers. Even though very few nurses and physicians described personal discomfort as a barrier, focusing on lack of time may mask other barriers that may be more challenging for practitioners to address such as feelings of frustration, a sense of futility or helplessness about how best to respond.

It is noteworthy that of the barriers described by respondents in this study, 51.0% were attributed to the abused women themselves (57.8% nurses; 36.1% physicians) suggesting that practitioners described fewer barriers related to their own behaviours. Their frustration with women choosing not to accept their advice or returning to partners after leaving, in and of itself, suggests the need for more education with respect to the complex dynamics of IPV.

As noted in Table 1, formal IPV training is not common and others have found that this kind of basic education is not the norm [28,29]. This reported lack of formal training points to potential curriculum gaps and the need for continuous learning opportunities in the work place. Both nurses and physicians indicated that they wanted to know how to introduce the topic and what to do if the woman discloses. Challenges in engaging women in such sensitive discussions have been noted by other investigators [13,14,30]. It is recognized that training can heighten sensitivity and enhance awareness. Hence, feelings of frustration expressed by both nurses and physicians accompanied by attitudes that appear to blame women may be consistent with low rates of formal training. Furthermore, it is not surprising that with the low rates of formal training, 30.4% of nurses had never heard a disclosure compared to only 5.0% of physicians (Table 1). However, recognizing that reports of formal IPV training were low for both nurses (37.0%) and physicians (40.8%), the stark differences in disclosures between nurses and physicians may be explained by role differences, patient behaviour, the nature of the patient interaction and the practice setting, especially in terms of safe and confidential spaces for these discussions. Nonetheless, this finding is alarming given the prevalence of woman abuse and that respondents were recruited from practice areas where IPV is likely to be encountered.

The seemingly greater emphasis on training by nurses requires further inquiry. This finding is interesting considering that the proportion of physicians (58.0%) and nurses (61.5%) who had no formal IPV training is similar. Many respondents, especially nurses, recommended training during their formal educational programs, continuing professional education, the opportunity to practice such skills and the opportunity to learn from the experiences of others. Greater emphasis on training by nurses may be related to role differences in client interactions depending on the discipline, and/or lack of experience with disclosure. Although training can result in greater knowledge, confidence and skill development, it is likely overly simplistic to suggest that training without periodic refreshers, other structural supports and organizational policies will result in significant practice changes [15]. This may be a practice issue that needs to be repeatedly addressed in a supportive practice setting to be understood and one that is very challenging to learn through simulated or theoretical experiences.

These findings suggest that in order not to blame women and to better address barriers to IPV inquiry, nurses and physicians need greater understanding of the complex dynamics and contextual factors that result in women continuing to deny abuse, not following through on intended actions or returning to abusive partners. Roberts et al. cite multiple stressors that abused women frequently encounter, including financial challenges, child custody battles, a sense of fear, altered social supports and feelings associated with the loss of emotional attachments with their abusers [31]. Furthermore, these authors suggest that abused women need to balance the rewards of leaving with the costs of this decision. Bennett and colleagues refer to the challenges that abused women often encounter when entering the justice system, such as a sense of confusion with the process, time delays, minimal information and the need for multiple appointments at a time when access to resources including child care, transportation and finances, are reduced [32]. Moreover, Bonomi et al. refer to both subtle and blatant pressures that abusers may direct towards abused women [33]. The impact of these cumulative stressors may be initially downplayed by abused women until experienced first-hand, resulting in the increased likelihood that women return to abusive relationships [31].

The impact on abuse disclosure and practitioner behaviour of legislation, that requires practitioners to contact child protective agencies when child abuse is suspected, is an area requiring further study. While further research about how best to address barriers to IPV inquiry faced by nurses and physicians is warranted, some actions in the workplace can be instituted. The potential challenges of collaborating across sectors can be minimized by developing strong relationships and an understanding of other sector roles and responsibilities. Providing training and regular updates, facilitating quick and easy access for consultations and the opportunity to discuss individual situations are important supportive strategies rather than leaving practitioners to face such decision points alone.

Overall, the nurses and physicians cited similar barriers and facilitators; however there were important differences in responses between the two disciplines. Some differences, such as language barriers, partner presence and lack of privacy, which were described more often by nurses, may be related to differences in role expectations and work environments between nurses and physicians. Similarly, because nurses are more likely to be employees, management support and agency policy are important factors for nurses when dealing with IPV. Knowing the types of barriers experienced by practitioners can influence the need for workplace policies and the development of pre-service and in-service training materials. Such resources can be customized by discipline, while customization by practice setting is an area for future study.

Our findings are consistent with previous studies of barriers and facilitators to asking about IPV, in particular barriers such as lack of provider education, frustration, lack of time and fear of offending a patient exposed to IPV [13,14,18]. It is disappointing that many of the barriers to abuse inquiry have not changed in the last 15 years. However, this finding suggests that approaches designed to assist practitioners in handling these commonly cited barriers may rest in the synergistic nature of the barriers and such approaches should not attempt to deal with barriers one at a time. A systems perspective that recognizes the complexity and inter-connectedness across barriers may yield more promising results.

In addition to corroborating the results of past studies, this study provides further insight into why some barriers have not changed. Lack of clinician confidence is not surprising, given the paucity of evidence-based interventions to which abused women can be referred [34]. IPV is a complex and multidimensional issue that is not solely the domain of the health care sector. Interprofessional and cross-sectoral collaboration is required. Expressions of professional challenges in being able to adequately meet the needs of abused women, and feelings of frustration and concerns about not knowing what to do if women disclose, suggest that more is needed to support practitioners. In order to navigate the complexities that surround asking about IPV, practitioners require specific personal skills and knowledge, access to community resources and work environments that encourage the development and sustainability of these skills. Mentoring, coaching and opportunities for debriefing and reflective practice are important supports for sustaining practitioner capacity for IPV inquiry.

Although barriers and facilitators were categorized, it is evident from the analysis that there is some overlap across categories. This suggests the need for comprehensive frameworks to explore further the many issues associated with IPV inquiry and the interplay across these issues. Multifaceted i.e., practitioner, workplace, community and patient-centred, intersectoral approaches are warranted. The complexities associated with IPV inquiry require action at many levels and failing to systematically address all levels may yield less than desirable results.

These findings highlight the need for on-going rigorous research to assist in identifying best practices from the perspective of both abused women and practitioners. In addition, the needs of all women should be considered when developing patient-centred approaches that are sensitive to race, ethnicity, socioeconomic status, religious/spiritual beliefs, age, ability and sexual orientation.

### Strengths and limitations of the study

This study has several strengths. Existing practice directories were used to randomly identify possible participants; responses from these participants provided rich data to explore the themes outlined above. The willingness of these respondents to provide detailed comments suggest that these practitioners were committed to IPV related issues. However, despite the high proportion of physicians (72.6%) who provided written comments among those who responded, only 32.8% (328) of physicians invited to participate in the study returned the survey. This may have limited the identification by physicians of perceived barriers and facilitators. However, while there is, in theory, a greater chance for bias when response rates are low, such response rates do not always mean biased responses [35]. Others have noted the challenges to obtaining high response rates to mailed surveys targeting physicians [36].

Furthermore, respondents may have been inclined to provide more socially acceptable and brief answers considering the sensitivity of the topic and the method of data collection, yet the volume and the richness of the written responses mitigate these limitations. Although the mailed survey offered respondents anonymity and a sense of privacy in which to share their perspectives and experiences without sanction, there was no opportunity for the researchers to seek clarification or further detail. The participants completed the open-ended questions without the benefit of interviewer prompts and without the stimulus of other participants. Future research would benefit from follow-up interviews which could provide further insights into the differences between nurses and physicians.

## Conclusions

IPV is a significant public health concern. This study identified barriers and facilitators to asking about IPV among Ontario, Canada nurses and physicians working in family practice, emergency medicine, public health, obstetrics/gynaecology and newborn care. Exploring why barriers to IPV inquiry continue to persist is important given the stigma associated with IPV, low disclosure rates and the impact of IPV on individuals, families and the larger community. A number of statistically significant differences between nurses and physicians were noted. The most frequently described barriers included lack of time and behaviours attributed to women living with abuse, such as denial and lack of action. The study respondents described professional supports such as the availability of social workers and access to community resources as facilitators. Although training was noted as a facilitator by both physicians and nurses, it was noted more often by nurses.

While these findings echo those of previous studies, this research provides new insights into the complex relationships among barriers and facilitators faced by physicians and nurses and why barriers to IPV inquiry have not changed in the past 15 years. This study also highlights the need for multifaceted strategies such as pre-service, service and continuous learning, practitioner supports, supportive work environments and workplace policies that collectively address individual, interpersonal, workplace and systemic issues faced by practitioners in addressing the challenges and complexities associated with IPV inquiry.

## Abbreviations

CAS, Children’s Aid Society; ED, Emergency department; IPV, Intimate partner violence; N, Nurse; P, Physician.

## Competing interests

The authors declare that they have no competing interests.

## Authors’ contributions

CEB assumed responsibility for the qualitative analysis and prepared the initial draft. IAG conducted the statistical analysis and vetted the qualitative analysis. HLM and CNW obtained study funding. All authors read and approved the final manuscript.

## Pre-publication history

The pre-publication history for this paper can be accessed here:

http://www.biomedcentral.com/1471-2458/12/473/prepub
